# Revisiting demographic processes in cattle with genome-wide population genetic analysis

**DOI:** 10.3389/fgene.2015.00191

**Published:** 2015-06-02

**Authors:** Pablo Orozco-terWengel, Mario Barbato, Ezequiel Nicolazzi, Filippo Biscarini, Marco Milanesi, Wyn Davies, Don Williams, Alessandra Stella, Paolo Ajmone-Marsan, Michael W. Bruford

**Affiliations:** ^1^School of Biosciences, Cardiff UniversityCardiff, UK; ^2^Parco Tecnologico PadanoLodi, Italy; ^3^Faculty of Agriculture, Università Cattolica del Sacro CuorePiacenza, Italy; ^4^Dinefwr, National TrustLlandeilo, UK; ^5^Independent ResearcherSwansea, UK

**Keywords:** cattle, *Bos taurus*, *Bos indicus*, phylogeography, adaptation, SNP array

## Abstract

The domestication of the aurochs took place approximately 10,000 years ago giving rise to the two main types of domestic cattle known today, taurine (*Bos taurus*) domesticated somewhere on or near the Fertile Crescent, and indicine (*Bos indicus*) domesticated in the Indus Valley. However, although cattle have historically played a prominent role in human society the exact origin of many extant breeds is not well known. Here we used a combination of medium and high-density Illumina Bovine SNP arrays (i.e., ~54,000 and ~770,000 SNPs, respectively), genotyped for over 1300 animals representing 56 cattle breeds, to describe the relationships among major European cattle breeds and detect patterns of admixture among them. Our results suggest modern cross-breeding and ancient hybridisation events have both played an important role, including with animals of indicine origin. We use these data to identify signatures of selection reflecting both domestication (hypothesized to produce a common signature across breeds) and local adaptation (predicted to exhibit a signature of selection unique to a single breed or group of related breeds with a common history) to uncover additional demographic complexity of modern European cattle.

## Introduction

Modern cattle comprise two species, *Bos taurus* and *Bos indicus*, both derived from the extinct aurochs (*B. primigenius*). The divergence between the two species has been dated ~250,000 years ago on the basis of mitochondrial DNA haplotypes (Bradley et al., 1996). The two species are interfertile and a number of hybrid breeds have been established (e.g., the American Beefmaster that was established from a Shorthorn × Hereford × Brahman cross). The details of cattle domestication have been a contentious, and arguments in favor of two or three domestication events have been put forward. The hypothesis arguing for two domestication sites suggests that taurine cattle were domesticated in the Fertile Crescent, likely from *B. primigenius*, while indicine cattle were domesticated in the Indus valley, likely from *B. primigenius namadicus* (Loftus et al., 1994; Troy et al., 2001; Lewis et al., 2011). The hypothesis arguing for three domestication sites adds a Northeast African center, that gave rise to the African taurine breeds deriving form *B. primigenius opisthonomous* (Wendorf and Schild, 1994; Payne and Hodges, 1997). Mitochondrial DNA evidence suggests that two main lineages were domesticated, one now typical of taurine breeds, and the other now typical of indicines (Loftus et al., 1994; Bradley et al., 1996). The domestication process is thought to have originated sometime between 8000 and 10,000 years ago (Ajmone-Marsan et al., 2010; Gautier et al., 2010; Lewis et al., 2011). Several potential routes have been hypothesized to explain the dispersal of taurine cattle from the Fertile crescent, mediated by Neolithic farmers, including alternative routes into Europe, occurring on at least two occasions (a Danubian route and a Mediterranean route Payne and Hodges, 1997; Pellecchia et al., 2007; Ajmone-Marsan et al., 2010; Taberlet et al., 2011). As aurochs only became extinct recently (Machugh et al., 1998; Beja-Pereira et al., 2006), it is possible that both taurine and indicine cattle crossed with their wild ancestor during their expansion out of the domestication centers, although the evidence supporting it is much debated (Troy et al., 2001; Gotherstrom et al., 2005; Beja-Pereira et al., 2006; Bollongino et al., 2008; Perez-Pardal et al., 2010; Edwards et al., 2011).

Several studies have addressed the population structure and phylogenetic relationships between taurine and indicine cattle breeds. Initially, studies were based on allozymes, mitochondrial DNA sequences, and microsatellites (Loftus et al., 1994; Medjugorac et al., 1994; Bradley et al., 1996; Machugh et al., 1998; Kantanen et al., 2000; Hanotte et al., 2002; Gautier et al., 2007) describing patterns of genetic variation in cattle, e.g., the two main lineages and differentiation between taurine and indicine breeds, albeit largely focusing on taurines. Nevertheless, such studies identified a clear differentiation between breeds from the two species, and between African and European taurines (Gautier et al., 2007). Interestingly, this handful of markers separated the African taurine breeds (e.g., Lagune, N'Dama and Somba) living in tsetse fly endemic regions from those that did not co-occur with these flies (e.g., Kuri and Borgou), suggesting that demographic signals among these breeds are strong and possibly related to properties of the environment they occupy. Recently, surveys of genome-wide genetic variation in many breeds have been carried out using SNP arrays (Bovine Hapmap et al., 2009; Mctavish et al., 2013; Decker et al., 2014). These more comprehensive analyses included tens of thousands of SNPs distributed across all chromosomes genotyped in several breeds from around the world. With these data, the differentiation between taurine and indicine breeds has been measured at approximately 10%, with approximately 5% for the divergence between African and non-African taurines (Gautier et al., 2010; Mctavish et al., 2013; Decker et al., 2014). Analyses using this type of large-scale data further identified a split between African longhorn taurines (N'Dama) and shorthorn taurines (e.g., Somba and Lagune—Gautier et al., 2010). Such analyses have also proved powerful enough to identify the mixed indicine/taurine genetic component of recently established hybrid breeds such as Santa Gertrudis and Beefmaster (Gautier et al., 2010; Mctavish et al., 2013).

Cattle are the most common large livestock species in the world; the global population size of which is approximately 1400 million animals (Felius et al., 2011; Taberlet et al., 2011). Among these, 1311 breeds have been recognized, 209 of which are now extinct (Rischkowsky and Pilling, 2007). Since domestication, cattle have spread around the world, with farmers selecting animals with desirable characteristics and establishing many breeds. This process, while not passive, was until recently slow and resulted in animals becoming adapted to the local conditions, e.g., feed types, local weather, and diseases (Wood, 1973; Cobb, 2006; Russell, 2007). The last two centuries saw a dramatic acceleration in the process of artificial/directional selection, largely as a consequence of the development of the breed concept, artificial insemination, and the development of statistical approaches used to estimate breeding values used to choose sires and dams for breeding purpose. This has limited the intercrossing between animals of divergent genetic background in order to increase or maintain breed purity, especially in dairy cattle [in beef cattle, conversely, cross-breeding is often used to exploit also non-additive genetic variation i.e., heterosis (Simm, 1998); still, pure lines for cross-breeding need to be selected and maintained]. Additionally, as a consequence of artificial insemination and marketing policies, few “champion” males with characteristics of interest have been used extensively to fertilize large proportions of females. The above developments have led to: (i) intense within-breed (or line) selection; (ii) importation/exportation of elite breeding stock to different environments/countries (migration); (iii) strong founder effect of “champion/top ranked” males both in breed formation or in already established breeds whose effective population size has thus been shrunken; (iv) larger genetic drift as a consequence of breed isolation. Generally, by trying to keep breeds pure, gene-flow among them has been reduced or even halted (unless done to improve breeds by upgrading Felius et al., 2011), and by using relatively few males to fertilize many females, inbreeding is increased (Taberlet et al., 2011). These approaches are used to manipulate the properties of a breed at the expense of the decrease in the genetic variability (Bulmer, 1971) which may be accompanied by a decrease in genetic health of the breed (e.g., reduction in fertility in Holstein cattle via the increase in frequency of deleterious variants through the process of inbreeding) (Pryce et al., 1997, 2004; Biffani et al., 2005). Overall, this change in farming practices resulted in a change from limited selection throughout much of the domestic history of cattle (~9800 years) to relatively stronger selection in the last ~200 years.

The relatively rapid distribution of cattle around the world over the last ~10,000 years suggests that populations have been potentially exposed to selective pressures deriving from the environmental variables that they had not been exposed previously, e.g., diseases occurring outside of the domestication centers (Hanotte et al., 2002; Felius et al., 2014; Xu et al., 2014). Consequently, while some cattle breeds represent recent introductions to new geographical areas such as the New World or Oceania, other breeds represent long established populations outside of the domestication center. The genetic makeup of such populations is a valuable source to understand the processes driving local adaptation, as the former represent genetic pools currently being shaped by local selective pressures (i.e., may serve as examples of selection in action) (Flori et al., 2012), while the latter represent established populations where the extant genetic variation reflects the outcome of local adaptation (Felius et al., 2014). In particular, long established local breeds are likely to represent the reservoirs of important genetic variation with adaptive potential that needs to be characterized (Taberlet et al., 2011; Felius et al., 2014) before it possibly disappears as a consequence of the widespread use of a handful of industrial breeds among others (Fao, 2007; Thornton, 2010; Herrero and Thornton, 2013; Perry et al., 2013).

Here, we collated a large-scale genome-wide dataset of SNP polymorphism in cattle breeds from around the world representing published taurine and indicine breeds, and new data for four European taurine breeds, all genotyped for a common set of ~35,000 SNPs in over 1300 animals. We used these data to characterize the distribution of genetic variation among cattle breeds around the world, and the genealogical relationships between these breeds. The dataset described here represents multiple breeds from various continents (e.g., multiple European and African taurines, as well as Asian and African indicines) providing a unique experimental set up that allows searching for signatures of selection that are consistent across multiple breeds from different continents. This approach enables to separate signatures of selection that may be breed specific from selection signals common for several breeds occurring in the same geographic region. We extend the published dataset with new SNP data to a case study where we compare white cattle breeds of conservation concern in the UK (White Park and Chillingham) with a candidate Roman ancestor, the Chianina, to help resolve the commonly held belief of a Roman origin of the Welsh White Park cattle (Felius et al., 2011).

## Materials and methods

### SNP array data

SNP array data was obtained for a total of 1346 animals representing 46 cattle breeds, and four species related to cattle (*B. gaurus*—Gaur, *B. javanicus*—Banteng, *B. gruniensis*—Yak, and *Bison bison*—Bison) used as outgroups (Table 1). The data comprises a combination of previously published datasets and new data collected for this study. The published data represents breeds published by Gautier et al. (2009); Gautier et al. (2010) and the Bovine HapMap project (Bovine Hapmap et al., 2009) genotyped with Illumina's BovineSNP50 v.1 and v.2 chip assay, respectively (Bovine Hapmap et al., 2009) (Table 1). Four new breeds were genotyped with the BovineHD Beach Chip for this project, namely, Welsh While Park (Dinefwr), Chianina, Romagnola and Chillingham. All SNP coordinates were converted to UMD3.1 bovine assembly (RefSeq: GCF_000003055.5). The datasets were merged using PLINK v1.7 (Purcell et al., 2007). The merged dataset was filtered to keep only individuals with 95% of their SNPs called and SNPs with 95% call rate across all samples. The SNPs and individuals that did not pass these filters were discarded. The total SNPs left in the dataset was 36,503 (data is available upon request from the authors). The data for each breed was phased with fastPHASE v1.4 (Scheet and Stephens, 2006).

**Table 1:** **Breed abbreviations and species identification**.

**Abb**	**BN**	**Tax**	***D***	***N***	**Ho**	***F***
ABO	Abondance	*Bos taurus*	2	22	0.308	0.089
ANG	Angus	*Bos taurus*	1	61	0.305	0.098
AUB	Aubrac	*Bos taurus*	2	22	0.301	0.112
BPN	Bretonne black pied	*Bos taurus*	2	18	0.317	0.064
BRU	French brown swiss	*Bos taurus*	2	18	0.288	0.150
BSW	Brown swiss	*Bos taurus*	1	24	0.280	0.171
CHA	Charolais	*Bos taurus*	1	20	0.314	0.071
CHL	Charolais	*Bos taurus*	2	26	0.320	0.054
GAS	Gascon	*Bos taurus*	2	22	0.305	0.100
GNS	Guernsey	*Bos taurus*	1	21	0.275	0.188
HFD	Hereford	*Bos taurus*	1	31	0.304	0.101
HO2	Holstein	*Bos taurus*	2	31	0.316	0.066
HOf	Holstein	*Bos taurus*	1	30	0.313	0.077
HOL	Holstein	*Bos taurus*	2	64	0.319	0.057
JE2	Jersey	*Bos taurus*	2	28	0.263	0.223
JEf	Jersey	*Bos taurus*	1	21	0.277	0.180
JER	Jersey	*Bos taurus*	2	28	0.263	0.223
LMS	Limousin	*Bos taurus*	1	44	0.309	0.086
MAN	Maine-Anjou (Rouge des Près)	*Bos taurus*	2	16	0.303	0.105
MAR	Maraichine (Parthenaise)	*Bos taurus*	2	19	0.318	0.059
MON	Montbeliarde	*Bos taurus*	2	30	0.299	0.116
NOR	Normande	*Bos taurus*	2	30	0.307	0.094
NRC	Norwegian red cattle	*Bos taurus*	1	21	0.317	0.063
OUL	Oulmès Zaer	*Bos taurus*	2	27	0.288	0.149
PMT	Piedmontese	*Bos taurus*	1	24	0.321	0.053
PRP	French red pied lowland	*Bos taurus*	2	22	0.325	0.039
RGU	Red Angus	*Bos taurus*	1	15	0.305	0.100
RMG	Romagnola	*Bos taurus*	1	24	0.291	0.141
ROM	Romagnola	*Bos taurus*	3	13	0.293	0.134
CHI	Chianina	*Bos taurus*	3	14	0.285	0.158
CIL	Chillingham	*Bos taurus*	3	16	0.026	0.924
W_P	White park	*Bos taurus*	3	15	0.245	0.276
SAL	Salers	*Bos taurus*	2	22	0.285	0.157
TAR	Tarine	*Bos taurus*	2	18	0.300	0.113
VOS	Vosgienne	*Bos taurus*	2	20	0.312	0.078
BAO	Baoule	*Bos taurus*	2	29	0.216	0.362
LAG	Lagune	*Bos taurus*	2	30	0.183	0.460
NDA	N'Dama	*Bos taurus*	1	25	0.209	0.381
ND1	N'Dama	*Bos taurus*	2	14	0.235	0.307
ND2	N'Dama	*Bos taurus*	2	17	0.237	0.299
ND3	N'Dama	*Bos taurus*	2	25	0.210	0.381
SOM	Somba	*Bos taurus*	2	30	0.217	0.359
BRM	Brahman	*Bos indicus*	1	25	0.190	0.440
GIR	Gir	*Bos indicus*	1	24	0.160	0.528
NEL	Nelore	*Bos indicus*	1	21	0.161	0.524
ZBO	Zebu Bororo	*Bos indicus*	2	23	0.240	0.292
ZFU	Zebu Fulani	*Bos indicus*	2	30	0.241	0.289
ZMA	Zebu from Madagascar	*Bos indicus*	2	30	0.194	0.427
BMA	Beefmaster	*hybrid*	1	24	0.328	0.031
SGT	Santa Gertrudis	*hybrid*	1	24	0.313	0.075
BOR	Borgou	*hybrid*	2	30	0.263	0.222
KUR	Kuri	*hybrid*	2	30	0.261	0.229
SHK	Sheko	*hybrid*	1	20	0.250	0.260
SIM	Simmental	*Bos taurus*	1	3	NA	NA
GBV	Gelbvieh	*Bos taurus*	1	3	NA	NA
OBB	North American Bison	*Bos Bison*	2	4	NA	NA
OBJ	Banteng	*Bos javanicus*	1	2	NA	NA
OGR	Gaur	*Bos gaurus*	1	4	NA	NA
OYK	Yak	*Bos grunniens*	1	2	NA	NA

*The three letter code used to identify the breed throughout the manuscript is shown (***Abb***), along with the breed name (***BN***) and the taxonomic status indicating the species name or whether the breed is a B. taurus x B. indicus hybrid (***Tax***), the data set's origin (***D***), namely Bovine Hapmap Consortium (1), Gautier et al., 2010 (2), this study (3), the breed's sample size (***N***), the observed heterozygosity (***Ho***), and the inbreeding coefficient (***F***)*.

### Population divergence

An analysis of population structure was carried out using the software Admixture v1.22 (Alexander et al., 2009), which uses a model-based estimation of indivdual ancestry for a range of prior values of K defined by the user. A cross-validation approach is used to determine the most likely number of populations (K) in the data. For each tested value of K, Admixture estimates the proportion of each individual's genotype deriving from each cluster. The values of K tested were in the range between 1 and 60 to accommodate potential population structure within breeds. For this analysis we excluded outgroup samples, breeds represented by fewer than 10 individuals, and SNPs with a minimum allele frequency lower than 0.01, linkage disequilibrium higher than 0.1 using a sliding window approach of 50 SNPs and a step size of 10 SNPs (Alexander et al., 2009). A principal components analysis was carried out using flashpca v1.2 (Abraham and Inouye, 2014) with default settings in order to investigate the ordinal relationships among breeds and individuals. Lastly, a NeighbourNet network was constructed using Reynold's distance among breeds using Splitstree v4.13.1 (Huson and Bryant, 2006), and a dendrogram using the same genetic distance and 100 bootstrap replicates to assess the statistical support of breed; clustering was performed using Phylip v3.69 (Felsenstein, 1993).

Within this framework, the Welsh White Park cattle breed was analyzed along the other cattle breeds to identify their relative position within the dendrogram and PCA explaining the similarities between cattle breeds. In particular, the common belief of a Roman origin of the Welsh White Park was addressed by comparing the position of this breed in relation to the Italian breeds, in particular the large Chianina breed, one of the oldest cattle breeds originating from the region of Valdichiana in central Italy, and Romagnola. Additionally, we estimated the relationships between Welsh White Park and other 17 breeds in the dataset using Treemix v1.12 (Pickrell and Pritchard, 2012) in order to determine the historical relationships between these breeds in terms of splits and migration (mixtures) between breeds. The breeds used for this analysis were Welsh White Park, the Italian Chianina, Romagnola (ROM), and Piedmontese, the British breeds Chillingham, Hereford, Angus, Jersey (JER), as well as five other non-African taurines (Holstein (HOL), Brown Swiss, Charolais, Normande, Vosgienne), the four African taurines Baoule, Lagune, Somba and N'Dama (NDA), and the indicine breed Brahman as outgroup. Treemix was run iteratively for values of the migration parameter between 0 and 12. The f index representing the fraction of the variance in the sample covariance matrix ($\hat{\text{W}}$) accounted for by the model covariance matrix (W) was used to identify the number of modeled migration events that best fitted the data (Pickrell and Pritchard, 2012).

### Demographic estimation

Recent demographic history was measured by the trend in effective population size (Ne) change over the last 8000 years using the software SNeP v1.0 and default settings (Barbato et al., 2015). Loci with missing data were excluded from this analysis, as well as SNPs with a minimum allele frequency (MAF) smaller than 5% (Sved et al., 2008; Corbin et al., 2012). Linkage disequilibrium (LD) was calculated between each pair of SNPs separated by a minimum distance of 5 kbp and maximum distance of 1 Mbp using Hill and Robertson's *r*^2^ (Hill and Robertson, 1968). LD values were grouped in 30 distances bins, such that each bin represented approximately the same number of pairwise LD estimates used to estimate Ne (Barbato et al., 2015). Within each bin the mean *r*^2^ was calculated and used to estimate *N_t_* = 1/(4 *f*(*c*)) (1/*r*^2^ − 1), where *f*(*c*) = *c* [(1 − *c*/2)/(1 − 2)^2^] (Sved, 1971); *c* is the linkage distance inferred from the physical distance between SNPs assuming 1 *Mb* ~ 1 *cM* and *N_t_* represents the effective population size estimate at *t* = 1/2*c* generations ago.

### Signatures of selection

**Figure 1:**
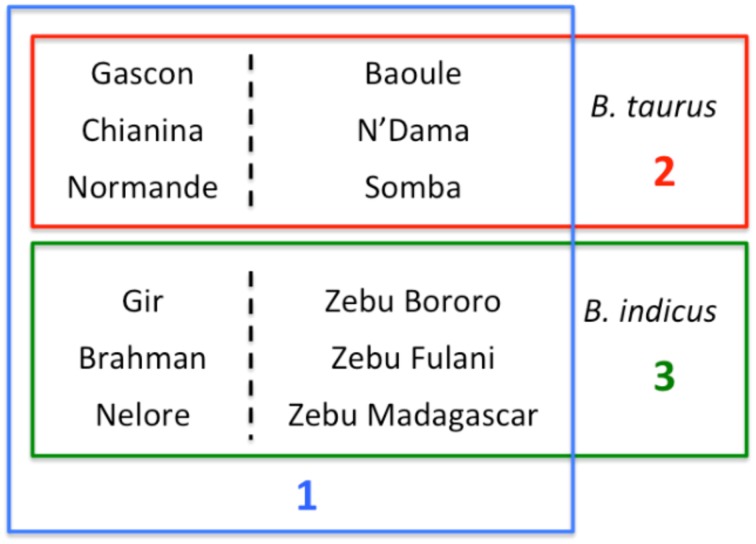
**Pairwise comparisons for analysis of selection**. Each box corresponds to one of the three types of pairwise comparisons carried out to detect signatures of selection. The blue box (comparison 1) indicates the comparison between four taurine and four indicine breeds used to detect signatures of selection between these breeds. The red box (comparison 2) shows the taurine breeds used in the comparison between African and not African breeds. The green box (comparison 3) shows the indicine breeds used in the comparison between African and not African breeds.

As the dataset analyzed here comprised *B. taurus* breeds from Europe and Africa, as well as *B. indicus* breeds from Asia and Africa, we sought to identify signatures of selection in a replicated manner (Figure 1). Initially we compared four taurine breeds (two European—Gascon and Chianina; and two African—Baoule and N'Dama) with four indicine breeds (two African – Zebu Fulani and Zebu Bororo; and the Asian Gir and Brasilian Nelore; Nelore was recently derived from Indian Ongole cattle, therefore, we broadly refer to these breeds as Asian indicine) to identify signatures of selection between *B. taurus* and *B. indicus*. Within *B. taurus*, we randomly chose three European breeds to compare against three African breeds in order to identify selection signatures specific to breeds in these continents. In the same way, for *B. indicus* we compared three Asian breeds against three African breeds (Figure 1). In contrast to previous studies, this experimental design enabled us identifying potential signatures of selection reflecting adaption to the local environment, instead of breed specific signatures that potentially reflect the breed's particular history.

The test of selection used was the cross population extended haplotype homozygosity test (XP-EHH) calculated for each SNP in the dataset as implemented in Selscan v1.0 (Szpiech and Hernandez, 2014). Selscan was used with default settings except for the maximum gap in bp allowed between SNPs, which was set to 400,000 to address the BovineSNP50 SNP density. For the comparisons between taurine and indicine breeds the following pairwise combinations were used: N'Dama (African taurine)/Gir (Asiatic indice), Gascone (European taurine)/Zebu Bororo (African indicine), Baoule (African taurine)/Zebu Fulani (African indicine) and Chianina (European taurine)/Nelore (Asian indicine). For the within-taurine comparison three pairwise tests were done between a European breed and an African breed, and for the within-indicine comparison three pairwise comparisons were carried out between an Asiatic breed and an African breed (Figure 1). For each pairwise comparison, the XP-EHH results where standardized for each chromosome separately, and the 5 and 95% quantiles of the standardized XP-EHH distribution for all markers where used as threshold to identify outlier SNPs, i.e., SNPs with XP-EHH values equal or smaller (more negative) or equal or larger (more positive) than the chosen thresholds. As multiple XP-EHH were carried out for each comparison (e.g., four comparisons between taurine and indicine breeds) this analysis was the equivalent of a replicated test. We took advantage of this design to identify SNPs under selection as those that showed for several pairwise comparisons an extreme XP-EHH with the same sign (e.g., all pairwise comparisons should an extreme positive XP-EHH).

For the between species comparison we defined the consistent SNPs under selection as those showing the same XP-EHH trend in at least three of the four pairwise comparisons. We did not restrict ourselves to only those SNPs where the four tests showed the same result to avoid excluding SNPs that failed to pass the 5% MAF threshold within a particular breed but which showed a consistent signature of selection across the remaining three comparisons. For the within species comparison of breeds occurring on different continents we identified consistent SNPs under selection as those showing an extreme XP-EHH with the same sign in each of the three comparisons made. The SNPs showing consistent signatures of selection across multiple pairwise comparisons where linked to neighboring genes using a window approach of 50 K base pairs, and a Gene Ontology analysis was carried out on these SNPs using Gorilla (www.cbl-gorilla.cs.technion.ac.il; Eden et al., 2009).

## Results

Of the 36,503 SNPs analyzed in 1345 animals representing taurine and indicine breeds, the observed heterozygosity varied between a minimum of 0.026 (Chillingham) and a maximum of 0.33 (the hybrid breed Beefmaster; Table 1). The comparison of expected heterozygosity (unbiased to sample size) between the taurine and indicine breeds was not significant, as well as the comparison between breeds in and out of Africa (all Welch *t*-test *p*-values > 0. 2). However, the average observed heterozygosity in taurine breeds was slightly larger than that in indicine breeds [0.28 standard deviation (sd) 0.05 and 0.20 sd 0.04, respectively]. The average expected heterozygosity across breeds in both datasets was similar, i.e., 0.34 sd 0.0001. The inbreeding coefficient (*F_IS_*) was significantly lower for taurine breeds (*F_IS_*: 0.18 sd 0.16) than for indicine breeds (*F_IS_*: 0.42 sd 0.11; Welch *t*-test *p*-value: 0.0011). Within *B. taurus*, the African breeds presented a significantly higher inbreeding coefficient than the non-African taurines (Welch *t*-test *p*-value: 1.54 × 10^−7^). Similarly, the non-African taurines also presented significantly lower *F_IS_* values than either of the groups of indicine breeds (Welch *t*-test *p*-value: 1.84 × 10^−5^ and 0.026, respectively). Among the non-African taurines, the Chillingham presented by far the highest inbreeding coefficient of the entire dataset (*F_IS_*: 0.92), on average five-fold larger than other taurines, and two-fold larger than the indicine breeds. The Welsh White Park is thought to be related to the Chillingham breed, but presented an observed heterozygosity (0.245) only slightly smaller than the average value observed in the other non-African taurines. The *F_IS_* value for Welsh White Park (0.27) was less than a third of that of Chillingham but still almost twice the average across the other non-African taurine breeds (0.14 sd 0.14). With the exception of Chillingham, the four breeds genotyped here presented typical observed heterozygosity and *F_IS_* values for European taurine breeds (Table 1). Removing from the analysis the breeds with samples sizes smaller than 20 did not change the results (results not shown).

### Admixture and genetic relationships

Admixture analysis between taurine and indicine breeds was initially run for values of the number of clusters (K) between 1 and 60. The cross validation (CV) statistic used to choose the most suitable number of clusters had its lowest value at *K* = 60. However, the shape of the CV curve suggested that higher K values may present lower CV. Consequently, we carried out runs of Admixture for larger values of K (e.g., 80). However, the CV values continued to reduce the larger the K numbers tested (SM Figure 1). While it may be possible that some family structure within breeds explains this trend, the substantially longer run time needed by the algorithm for such K values combined with the small decrease in CV suggests that clustering solutions larger than K ~60 potentially represent spurious results (a similar observation was made by Mctavish et al. (2013) who only achieved marginal increases on the likelihood scores using structure in their cattle dataset for values of K beyond 3).

**Figure 2:**
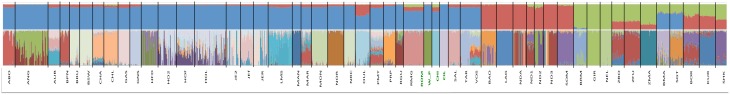
**Analysis of Population Structure**. Results of the analysis of population structure conditioning the dataset to 3 clusters (top row) and to 51 clusters (bottom row). Each animal is represented by a straight bar that is colored. The amount of a color reflects the individual's proportion of genetic variation originating in the cluster of that color. Each breed is labeled in the center of its box on the bottom of each plot. In the top row the non-African taurine breeds are labeled in blue, the African taurine in red and the indicine (both African and not African in green). The new datasets produced for this project are highlighted in green.

The hierarchical clustering analysis with Admixture showed that the largest difference for *K* = 2 was between taurine and indicine breeds. Conditioning the data to *K* = 3 separated the taurine breeds with an African origin from the non-African, suggesting a large differentiation between taurine breeds in these two groups of samples (Figure 2). Contrastingly, for values up to *K* = 10, the African indicine breeds presented a remarkably similar distribution of genetic variation to that of the Asian indicine breeds, which did not form a separate cluster. Nonetheless, the African indicine breeds consistently presented a distinctive proportion of their genotype representing taurine ancestry throughout the analysis (a pattern almost entirely absent in the breeds of Asian origin). The *B. taurus*/*B. indicus* hybrids show a genetic make-up consisting of non-African taurine and *B. indicus* genetic components (Beefmaster and Santa Gertrudis), or African taurine and *B. indicus* (Borgou, Kuri, and Sheko).

**Figure 3:**
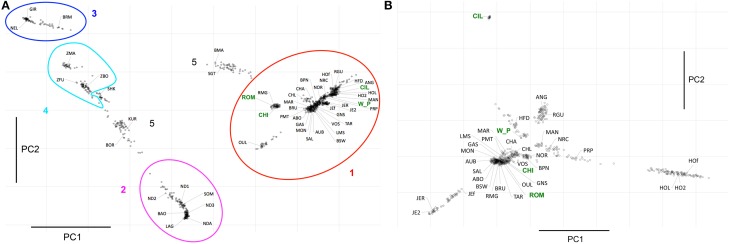
**Principal Component Analyses. (A)** This plots shows PC1 and PC2, which together explain ~15% of the variance in the dataset. The ellipses represent: Red=non-African taurine, Purple=African taurine, Dark Blue=Asian indicine, Light Blue=African indicine. Three letter codes refer to the breed abbreviations (Table 1). **(B)** Plot showing PC1 and PC2 of the analysis of the non-African taurine breeds, which together explain ~6.5% of the variance in that dataset. The new datasets produced for this project are highlighted in green in both **(A,B)**.

The Admixture analysis was complemented with a principal component analysis (Figure 3). The first principal component (PC1) explained ~10% of the total variation in the data and separated taurine form indicine breeds. Besides of PC1, the only other component explaining a substantial part of the variance was PC2 (~5%), which separates the African taurine from the remaining taurine, as well as the African indicine from the Asian indicine breeds. PC3 to PC7 explained between less than 2 and 1.4% of the variance, while the remaining components explained less than 1% of the total variance, thus were not taken into consideration. PC3 separated Holstein from the remaining taurine breeds. PC4 identified a group of British breeds formed by Angus, Red Angus and Hereford, and PC5 identified a component of variation specific to Hereford separating it from the other taurines. PC6 identified the Chillingham cattle as well as the Jersey breed, and PC7 identified a specific component of variation to Chillingham separating it from the remaining taurine breeds. The combination of PC1 and PC2 identify the major groups of individuals described here in terms of species and geographic component (Figure 3A) with the hybrid breeds occurring between these. As expected from the Admixture analysis, the breeds Kuri, Borgou, and Sheko are placed with these principal components between the group of African taurine and indicine. Similarly, Beef Master and Santa Gertrudis are placed between the group of non-African taurine and indicine breeds, although from this analysis it is not clear whether their indicine genetic component is more similar to African indicine or Asian indicine.

**Figure 4:**
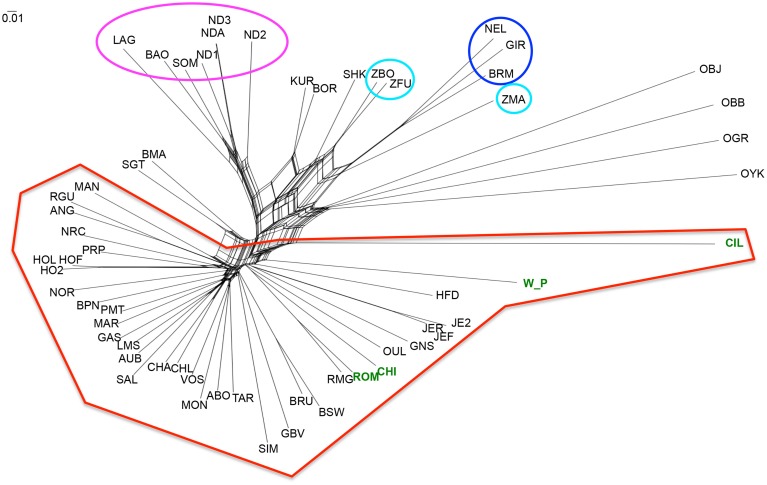
**NeighbourNet depicting the relationships between cattle breeds**. Three letter codes refer to the breed abbreviations (Table 1). The groups marked with ellipses in Figure 3A were marked with the same colors here as well. The new datasets produced for this project are highlighted in green.

The methods described above identified differences between breeds but did not allow us to determine how these relate to each other. We therefore estimated a NeighbourNet network with these breeds using Reynold's distance. The torso of the network shows multiple pathways of connection between breeds reflecting the relatively recent divergence between many populations (Figure 4). However, the topology of the network resulted in the pattern expected from the previous analyses (Admixture and PCA), where a clear separation between taurine and indicine occurs, as well as the distinct separation between the African taurine breeds from the non-African taurines. Similarly to the PCA and in contrast to the Bayesian approach of Admixture, the network also resolved the African indicine breeds separately from their Asian counterparts. The dendrogram depicting the clustering of breeds using Reynold's distance recovered the grouping of breeds identified in the PCA with most branches (83%) supported with high statistical support (bootstrap values higher than 70%; SM Figure 2). The clustering of the outgroups was not well resolved, except for the grouping between bison and yak. The indicine breeds clustered together with the Asian indicine showing a greater similarity to each other, and then to the Madagascan zebu. The two African indicine clustered together and then clustered with the remaining indicines. The taurine breeds separated into two clusters, one representing the African taurines and the other the non-African taurines. The hybrids Beefmaster and Santa Gertrudis clustered together within the non-African taurine group reflecting their larger taurine genetic component. Contrastingly, the African hybrids (Sheko, Borgou, and Kuri) clustered with the indicine breeds suggesting a higher indicine genetic component in their genotype.

**Figure 5:**
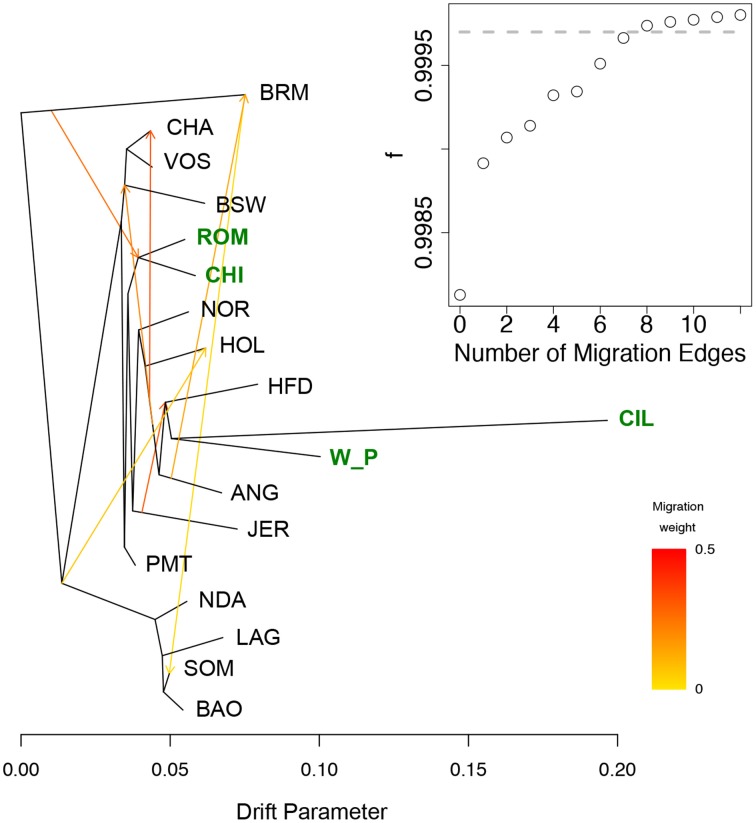
**Phylogenetic network of the inferred relationships between 18 cattle breeds**. The phylogenetic network inferred by Treemix of the relationships between Welsh White Park cattle breed and 17 other breeds is shown with Brahman (BRM) as outgroup. Migration edges between breeds are shown with arrows pointing in the direction toward the recipient breed of the migrants, and colored according to the ancestry percent received from the donor breed. The new datasets produced for this project are highlighted in green. The inset shows the f index representing the fraction of the variance in the sample covariance matrix ($\hat{\text{W}}$) accounted for by the model covariance matrix (W), as a function of the number of modeled migration events; the gray dashed line marks the number of migrant edges beyond which the f statistic asymptots.

A comparison between the Welsh White Park cattle and the other British breeds (Chillingham, Hereford, and Jersey) and Italian breeds (Chianina, Romagnola, Piedmontese) was carried out to establish whether the UK white cattle have a genetic link with Roman white cattle, as is popularly believed. The Principal Components Analysis of this datasets identified the White Park breed clustering near the other British breeds (i.e., HFD, ANG, RGU), instead of closer to the Italian breeds (Figure 3B), as would be expected if White Park cattle had a strong Italian genetic component due to its speculated Roman origin. Similarly, in the dendrogram, the Italian breeds clustered together and separately from the Welsh White Park and Chillingham breeds, the latter two not clustering with any of the other taurine breeds suggesting their genetic distinctiveness. The Treemix analysis revealed a similar topology with the Welsh White Park and Chillingham clustering together and then clustering to Hereford and Angus, rather than clustering with the Italian breeds (Figure 5, SM Figures 3, 4). Adding up to seven or eight migration edges to the tree topology improved the amount of the variance explained by the phylogenetic model, albeit marginally (f statistic ~ 0.99965; Figure 5; Pickrell and Pritchard, 2012). The model without migration edges already presents an f statistic of 0.9981 (Figure 5 inset), a value above the threshold described previously, and above which the phylogram was not better explained by adding additional migration events (Pickrell and Pritchard, 2012; Decker et al., 2014). However, more migration edges did not seem to further increase this variance as the f statistic reached an asymptote between 7 and 8 migration edges (Figure 5 inset). The largest increase in variance occurred by adding the first migration edge to the graph from Brahman to the ancestor of Chianina and Romagnola, consistently with the Admixture results that suggest these Italian breeds have a minor (~10%) indicine genetic component. With the exception of two edges linking Brahman, Angus and Somba, the remaining migration edges were between taurine breeds. All migration edges had a weight less than ~0.2, with the exception of those from Jersey into Hereford, and from the ancestor of Hereford, White Park and Chillinghan, into Charolais, both of which have a weight above 0.4, suggesting the source population made a substantial genetic contribution to the recipient breeds.

### Demographic history

**Figure 6:**
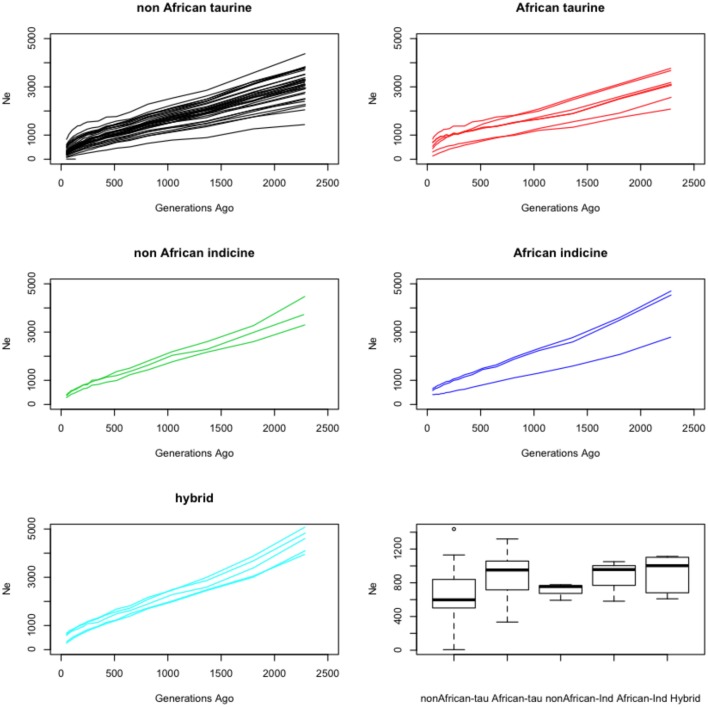
**Demographic history reconstruction**. Each plot shows the trend in effective population size for a set of cattle breeds between ~50 and ~2300 generations ago. The bottom right graph shows boxplots for the harmonic-mean of the effective population sizes trends estimated with SNeP for the various groups of cattle breeds analyzed.

The trend in historic effective population sizes for each breed was estimated using the distribution of linkage disequilibrium across the genome. All breeds showed a declining effective population size over the last ~2000 generations (i.e., approximately 8000 years assuming an average generation length of 4 years throughout these species history; Figure 6). Interestingly the African breeds showed an average higher effective population size for each species than the non-African breeds. Consistent with their hybrid nature, the five hybrid breeds presented a larger effective population size, probably reflecting the artificial increase in heterozygosity deriving from the admixture event (Figure 6).

### Signals of selection

A replicated approach using XP-EHH was used to identify consistent SNPs showing signatures of selection in the dataset (Figure 1). Four pairwise comparisons between taurine and indicine breeds were carried out resulting in 10,150 SNPs that passed the MAF threshold within population and which were tested in at least three out of the four pairwise comparisons. Among these SNPs, 3029 (~30%) showed consistent signatures of selection in the taurine breeds and 2385 (~24%) in the indicine breeds. The comparison between African and non-African indicine breeds resulted in 9388 SNPs tested, out of which 1768 (~19%) showed signatures of selection specific to the Asian breeds and 2049 (~22%) showed signatures of selection specific to the African breeds. For the comparison between taurine breeds, a similar number of SNPs was tested, 9938 SNPs. Of these 1872 (~19%) showed signatures of selection in European cattle breeds and 1739 (~17%) in African breeds. The SNPs showing consistent signatures of selection were linked to neighboring genes that occurred up to 50,000 bp downstream or upstream of the SNP position. No significantly enriched GO categories were found for any of the sets of breeds analyzed here. However, some patterns were observed, e.g., for the candidate SNPs under selection in Asian indicine several genes related to immunity were identified, e.g., LIPH (involved in platelet aggregation), Tespa1 (involve in thymocyte development), CCL14 (activates monocytes) (SM Table 1). However, interestingly, several RNA types were identified for several of the groups of breeds tested (Table 2). For the comparison between taurine and indicine breeds 6 snoRNA and 10 snRNA were identified among the taurine breeds, and two miRNA and 1 snoRNA in the indicine breeds. For the comparison between Asian and African indicine, a total of three miRNA, 1 snoRNA and 2 snRNA were identified for the Asian breeds and none for the African, while for the taurine comparison, the opposite was true, i.e., 1 snoRNA and 1 snRNA were identified for the African breeds and none for the European breeds. Overall this suggests that a substantial amount of selection signature occurred near non-coding RNAs that modify transcription of genes or of other RNAs.

**Table 2:** **Classes of molecules under selection**.

**Molecule Class**	**Taurine vs. Indicine**	
	**Taurine**	**Indicine**
Protein Coding	15	14
RNA (mi, sno, sn)	0,6,10	2,1,0
rRNA	0	0
Pseudogene	1	1
	Indicine	
	Asian	African
Protein coding	46	2
RNA (mi, sno, sn)	3,1,2	0
rRNA	3	0
Pseudogene	3	0
	Taurine	
	European	African
Protein coding	5	1
RNA (mi, sno, sn)	0	0,1,1
rRNA	0	0
pseudogene	0	0

*DNA sequences coding for molecules in close proximity to SNPs under selection were grouped as Protein Coding, RNA types (mi, sno, and sn), rRNA and pseudogenes. The number of SNPs under selection close to each of these classes is shown for each of the three analyses presented*.

## Discussion

This dataset represents one of the most comprehensive analyses of *B. taurus* and *B. indicus* cattle breed genetic data. Overall a higher observed genetic variation was observed in taurine breeds that originated or derived from European cattle breeds (~0.29), than in the other cattle breeds. Both African taurine and indicine breeds presented an intermediate level of observed heterozygosity (~0.22 in both sets) and the Asian indicine breeds presented the lowest observed heterozygosity (~0.17). While the observed difference could have a generic demographic explanation, e.g., a larger effective population size in the taurine breeds of European descent, such pattern is not consistent with what has been described with other types of molecular markers such as microsatellites (Rothammer et al., 2013). The most likely explanation for this pattern is ascertainment bias (Gautier et al., 2009; Matukumalli et al., 2009). In particular, for those breeds not included in the panel of breeds used to identify SNPs in the BovineSNP50 chip assay, minimum allele frequencies are expected to be substantially lower and the genetic variation captured with the chosen SNPs may not be reflective of the true underlying genetic variation in those breeds. In this case, SNP discovery was based on the genome sequence reads of five taurine breeds (Holstein, Angus, Limousin, Jersey, Norwegian Red) and one indicine (Brahman) breed compared to the reference genome of a Hereford cow (Bovine Hapmap et al., 2009). Ascertainment bias deriving from the design of this SNP array was previously described for a subset of the dataset analyzed here, where up to 30% of the SNPs had a MAF > 0.3 in taurine breeds, while only 19% of the SNPs had an equivalent MAF in indicine breeds (Nelore, Brahman, and Gir) (Bovine Hapmap et al., 2009). Similarly, the analysis of Holstein, Angus and Brahman samples carried out with a subset of the SNPs in the BovineSNP50 chip assay ascertained in each of the breeds separately, showed how Brahman animals presented a lower heterozygosity using the taurine SNPs (*p*-value < 0.05 and *p*-value = 0.07, respectively) than using the indicine SNPs (Neto and Barendse, 2010). Contrastingly, in the same analysis, for the Holstein and Angus derived SNPs, the taurine samples presented an observed heterozygosity typical to that of any other taurine breed. Furthermore, as no African taurine breeds and most indicine breeds were not included in the SNP ascertainment, the observed levels of genetic variation are likely to be an underestimate of the true genetic variation in these breeds (Matukumalli et al., 2009), and the associated inbreeding coefficient may consequently be an overestimate of the real ones. Thus, comparisons of genetic diversity discussed here are only likely to be meaningful within major genetic groups.

Among all cattle, the British Chillingham breed possessed unusually low observed heterozygosity (0.026) and an extremely high inbreeding coefficient (0.92). The Chillingham occurs in Northumberland, UK, and for the last ~300 years has lived in a feral state with almost no human intervention and no immigration (Visscher et al., 2001). Additionally, this breed passed through a bottleneck in 1947 where the herd reduced to five males and eight females. Previous analysis of genetic variation using 25 microsatellites identified only one marker with polymorphism (Visscher et al., 2001). Additionally, sequences of the full mitochondria of eight animals (sequencing depth ~2935x using next generation sequencing) revealed a single haplotype in the herd that differentiated them from other taurine breeds by three mutations, and one mutation (likely to be a recurrent mutation) that related them to indicine breeds and yak (Hudson et al., 2012). These studies suggest that Chillingham probably survives because the population has purged most deleterious mutations. The results shown here represent the largest study of genetic variation in this breed so far and did find similarly low levels of genetic variation in the breed (~900 polymorphic SNPs out of ~35,000). An extended analysis including more individuals and genetic markers in this breed may identify genetic variation of evolutionary importance not captured in this study, as well as shedding light on the distribution of homozygous and heterozygous tracts in the breed that could be used to monitor its genetic health.

### Population structure

The analyses of population structure easily identified the taurine and indicine genetic components in the dataset (Figure 2). However, when conditioning the number of clusters in the data to *K* = 2, the African taurine cattle presented a genetic composition similar to that of the American hybrid breeds Beef Master and Santa Gertrudis, the latter two are breeds of recent origin (less than 100 years old). Increasing the number of clusters to *K* = 3 clearly separates the African taurines in their own cluster, suggesting that despite the strong non-African taurine and indicine genetic component (as seen for *K* = 2), African taurines (both longhorn and shorthorn) have a substantial third genetic component specific to Africa which may derive from cross breeding with local aurochs (Mctavish et al., 2013). Consistent with previous observations, the African taurine N'Dama (ND1 and ND2) and Somba (SOM) breeds presented a minor indicine genetic component (Gautier et al., 2010). Among the hybrids, Beefmaster (BMA) and Santa Gertrudis (STG) presented a major genetic component of non-African taurine origin and the remaining of indicine origin, in accordance with their breed formation. Beefmaster and Santa Gertrudis are both hybrid breeds of North American origin resulting from the cross between European taurine cattle of British origin (Hereford and Shorthorn) and Brahman cattle (Mctavish et al., 2013). On the other hand, the remaining three hybrid breeds, the Borgou, Kuri, and Sheko present a genetic make-up of African taurine and indicine origin, with the first two having a somewhat larger taurine proportion than the Sheko breed. Among the taurine breeds, the Moroccan Oulmès Zaer (OUL) breed of unknown origin, presented approximately ~3/5 of its genotype of non-African taurine type and the remaining of African taurine type (Figure 2), consistent with previous results suggesting an influence of African longhorn taurine into the otherwise European taurine genetic background of this breed (Gautier et al., 2010; Decker et al., 2014). Interestingly, the non-African taurine cattle presented around 10% or less of their genetic make-up showing African taurine ancestry (Figure 2), possibly deriving form cross Mediterranean movement of cattle during the last millennia (Dürst, 1899; Do Vale, 1907; Felius et al., 2011). A further indicine genetic component was also detected in the French Charolais and Gascon breeds (<5%), and the Italian Romagnola and Chianina breeds (~10%). These results support observations by Decker et al. (2014) that suggest indicine admixture in European taurine breeds (the bulk of non-African taurines in this study) is rare instead of widespread (Mctavish et al., 2013). The genetic variation of African taurine is clearly distinct from that of other cattle breeds, and the PCA analysis suggests that the three main cattle groups (African and non-African taurine, and indicine) are almost as different from each other. While these results do not provide evidence for or against the hypothesis of a third domestication event in Africa (Felius et al., 2011), it suggests that African taurine cattle present a unique characteristics in their gene-pool.

The analysis of population structure using Admixture resulted in asymptoting CV values reflecting problems in identifying the true number of clusters in the data, possibly due to the presence of family structure within the breeds, or relatively low divergence between the estimated clusters. Thus, we also carried out principal components analysis, and as expected from the problems with Admixture, there is relatively low variation among cattle groups, i.e., the largest principal component (PC1) only explains ~10% of the variance in the dataset (separating taurine form indicine breeds), while PC2 explains ~5% of the variance (separating African from non-African taurines). Consistent with the Admixture result, the combination of PC1 and PC2 places the hybrid breeds Kuri, Borgou and Sheko between the African taurine and African indicine clusters, with the Sheko much closer to the indicine cluster reflecting its larger indicine background than Kuri and Borgou. The American Beef Master and Santa Gertrudis are placed between the non-African taurine and Asian indicine, but somewhat closer to the taurine cluster reflecting the larger taurine component of their genetic variation.

Lastly, the NeighbourNet analysis and the dendrogram identify the Madagascan zebu as more similar to the Asian indicine than to the other two African indicine. While this placing may be biased due to the ascertainment bias in the SNP array, it is tempting to suggest that the Madagascan population share a more recent history with Asian indicine breeds than the continental African breeds (e.g., a more recent establishment of the Madagascan population).

### Roman origin of Welsh White Park

Within the United Kingdom, traditional knowledge suggests that the Welsh White Park were brought to Britain by the Romans (Felius et al., 2011). While there is no evidence of the Romans having introduced cattle to Britain (Visscher et al., 2001), PCA places the White Park almost in the middle of the group of non-African taurine breeds (Figure 3A), consistently with the results by Decker et al. (2014) based on genotypes from five individuals. In particular, a comparison between Welsh White Park and the three Italian breeds in this dataset, Chianina, Romagnola and Piedmontese, revealed that the Italian breeds are more closely related to each other than to White Park, and the Welsh breed is more closely related to the other British breeds (Figures 3B, 5). Nevertheless, while this suggests that Welsh White Park are not similar to the Italian breeds it still does not answer the question regarding their origin. Both the NeighbourNet (Figure 4) and the phylogenetic network (Figure 5) show that the Welsh White Park and Chillingham cluster near each other (Figure 4). Such observation could be the outcome of genetic drift or inbreeding (or an interplay of these two parameters) in these breeds driving the divergence of their allelic frequencies (particularly in the case of Chillingham). Consistently with this hypothesis, the phylogenetic network analysis modeling genetic drift suggests that Welsh White Park and Chillingham have been through substantial drift in comparison to other taurines (Figure 5). Additionally, the Treemix analysis suggests that no migration events connect either the Welsh White Park or Chillingham with any of the Italian breeds, unless an additional edge that marginally increases the f-statistic by 0.01% (i.e., from 99.96 to 99.97% of the variance explained) is added; however, that additional migration edge is from Welsh White Park into Romagnola (contrary to common believe of an Italian ancestry of White Park) and has a weight near 0 (SM Figure 4) indicating that a very small fraction (if at all) of the Romagnola allelic variation may be of Welsh origin. While such idea could be appealing to some, it does not necessarily reflect a real historic event as the inferred edge provides an almost negligible contribution to the variance in the phylogram and corresponds to a minimal contribution of Welsh allelic variants into Romagnola. Additionally, the question of whether the inferred patterns of migration by Treemix are somehow dependent on the data analyzed could be raised. Further studies on the genetic history of these iconic breeds should include additional breeds such as the Podolian Hungarian Gray, or breeds close to the domestication center in order to better characterize the origin of this animals.

### Demographic history

The demographic history of a population can be traced using the distribution of LD across the genome (e.g., Hill and Robertson, 1968), and if the cattle domestication event was marked by a strong bottleneck, it is expected that such a pattern should be visible with this dataset. However, it is important to note that the analyses presented here might provide a biased estimate of the true extent of demographic history, as LD calculated over SNP array markers is expected to decay at a lower rate than when the same estimates are obtained from whole-genome sequence data (Qanbari et al., 2014).

All the breeds analyzed present trends marked by a decline in their effective population size during the last ~2000 generations (Figure 6). Assuming an average generation length of 4 years, that suggests a constant decline across all breeds since the onset of the domestication process approximately 8000 years ago (Gautier et al., 2010). These results are consistent with observations describing a general decrease in breeds such as Angus, Holstein, Hereford, N'Dama and Brahman over the same period of time (Gautier et al., 2007; De Roos et al., 2008; Flury et al., 2010). Interestingly, overall, the harmonic-mean effective population size of the non-African taurine breeds was smaller than that of the non-African indicine, but both of those means were much smaller than that of the African taurines and indicines (Figure 6). These results suggest that either crossing between African taurine and indicine breeds has increased the effective population size in these breeds by moving genetic variation between breeds, and/or that more benign management practices in Africa have resulted in a milder reduction in effective population size. Although the second hypothesis cannot be ruled out, consistent with the first hypothesis, the hybrid breeds present overall a higher average effective population size.

### Signatures of selection

The unique nature of the data presented here provided us with a potentially powerful statistical framework to detect signatures of selection. In contrast to other studies, we examined multiple breeds for each species, accounting for their distribution in and out of Africa to search for signatures of selection in the equivalent of a replicated experimental design, where instead of identifying selection in a particular breed, we used several breeds of each species in each continent as biological replicas. The test used is an extension of Sabeti's extended haplotype homozygosity (Sabeti et al., 2002), which is based on the principle that a site under positive selection will rapidly increase in frequency, bringing neighboring variants to a high frequency in a process called hitchhicking (Smith and Haigh, 1974), thereby removing genetic variation around the site under selection (a selective sweep) and leaving a track of extended homozygosity. The magnitude of the effect of the selected site on the neighboring variation largely depends on how strong and recent the selective pressure is. If the process is slow, because selection is weak, recombination events separate the selected site from neighboring variants breaking down linkage and consequently resulting in a short area of extended homozygosity (Sabeti et al., 2002). On the contrary, if the process is fast because selection is strong, the area of extended homozygosity will be large, as there is not enough time for recombination to break down linkage. Consequently, identifying old signatures of positive selection using the EHH or any of its derived versions (e.g., XP-EHH) is rather difficult as the expected pattern of linkage disequilibrium is likely to have disappeared (Sabeti et al., 2007; Chen et al., 2010). However, despite the ancient divergence between *B. taurus* and *B. indicus*, i.e., approximately 250,000 years old (Bradley et al., 1996; Bovine Hapmap et al., 2009), a consistent signature of selection was found for several thousands of SNPs (5385 SNPs) probably reflecting relatively recent selective events rather than ancient ones.

Consistent with the expectation for the comparison between breeds in both species, a similar number of SNPs in each species showed signatures of selection (i.e., 3029 in taurine and 2385 in indicine). For the African vs. non-African comparison of taurine breeds 1872 of the SNPs under selection were on European breeds and 1739 were in African breeds. In contrast, for the comparison of African vs. non-African indicine breeds, 1768 of the SNPs under selection were on the Asian indicine breeds and 2049 were in the African breeds. The difference between the comparison of taurine breeds and indicine breeds in and out of Africa may reflect the shorter period of time that the later have spent in Africa since their introduction to that continent. The first domestic cattle to have entered Africa were taurines approximately 7000 years go (Payne and Hodges, 1997; Hassan, 2000; Felius et al., 2014). These domestic animals probably entered via a route crossing through Egypt and moving into northern Africa. In contrast, indicine breeds entered Africa much later via routes leading to East Africa via Egypt some time between ~3500 and 2500 years ago (Payne and Hodges, 1997; Hanotte et al., 2002). The timing of these migration events into Africa implies that taurine breeds have been in Africa between two and three times longer than indicine breeds.

At least three non-exclusive hypotheses can be derived from the taurine colonization history of Africa. Firstly, African taurine have become clearly differentiated from other non-African taurines as genetic drift exacerbated differences between the taurine propagules that spread from the domestication center into Europe and Africa. Secondly, African taurines had a longer time to hybridize with local aurochs, as possibly was the case for the European taurines, which hybridized with European aurochs (Decker et al., 2014). Consequently, African taurines are expected to carry unique genetic variation (deriving from local aurochs) that differentiates them from non-African taurines, as African aurochs were likely to genetically differ from European aurochs due to isolation by distance (Perez-Pardal et al., 2010; Mctavish et al., 2013). Thirdly, by having spent a longer time in Africa, African taurines are expected to have had a longer time to adapt to the local environment and although some of their signatures of local adaptation may have dissipated, they may have left a trace of genetic identity that helps differentiating African taurine form other cattle breeds. In contrast, *B. indicus* was introduced to Africa approximately ~2000 years ago, and during this short period of time African indicine breeds may have not had enough time to differentiate from other indicine breeds, as much as the African taurines did. However, as the presence of indicine breeds in Africa is recent it is reasonable to expect that these carry more signatures of selection than African taurines (~22 vs. ~17%, respectively).

The analysis of selection carried out here sought to identify SNPs under selection using the equivalent of a replicated experiment. For that purpose it was necessary that several pairwise comparisons identified the same SNP under selection showing the same sign in the XP-EHH analysis (e.g., a SNP showing selection for the European taurine variant in the three pairwise comparisons). The rationale for this is that genetic drift can cause extreme differences in allele frequencies between populations, however, the chance that the same allele changes allele frequency in the same direction in multiple populations is low (e.g., A gets fixed by chance in the three replicates). While our approach is expected to be robust as it combines the power of the track of linkage disequilibrium left after positive selection and its replicability across populations occupying similar environments (e.g., three European breeds tested against three African breeds), it is possible that weaker signatures of selection were not identified. Nevertheless, it is interesting to note that for the pairwise comparisons where several genes were identified to be in close proximity to the SNPs under selection some chromosomes seems to be more frequent under selection than others. For example, among the Asian indicine, eight genes occurred on the first chromosome, and 11 on the 19th chromosome (SM Table 1), while three out of the four genes identified among the European taurine breeds occurred on the 10th chromosome. Lastly, it is expected that short term adaptive processes may be mediated through modifications of regulatory pathways (Karim et al., 2011; Visscher and Goddard, 2011; David et al., 2013; Furusawa and Kaneko, 2013). Consistently with such hypothesis we find that several SNPs under selection occur in close proximity to RNA coding sequences involved in transcription regulation (e.g., miRNAs) and modification of other RNAs (e.g., snoRNAs).

The domestication of cattle in Eurasia had profound implications for society (Ajmone-Marsan et al., 2010; Groeneveld et al., 2010; Felius et al., 2014). Cattle are one of the major livestock species in the world and represent a large assemblage of widespread breeds as well as locally adapted breeds (Ajmone-Marsan et al., 2010; Felius et al., 2011, 2014). Studying the genetic resource of cattle breeds around the world is an on going work (e.g., Bovine Hapmap et al., 2009; Gautier et al., 2010; Mctavish et al., 2013; Decker et al., 2014) and approaches like the ones used here show the power that multiple comparisons have to derive particular trends and patterns that have characterized these breeds' histories. Additionally, identifying gaps representing regions where cattle has not yet been studied, as well as breeds that have not yet been genetically characterized is of utter importance to identify the genetic variation with adaptive value (e.g., local adaptation) and that should be the target of conservation efforts. Finally, whether Welsh White Park cattle were introduced to Britain by the Romans seems unlikely with the data described here, but cannot be entirely ruled out. The current results suggest that the breed presents a distinct genetic variation possible reflecting its ancient origin, but without some sort of calibration for the molecular phylogeny, an Approximate Bayesian computation approach that estimates times of divergence between breeds, or its comparison to local breeds close to the domestication center, this question is left partially unanswered (Csillery et al., 2010a,b; Sunnaker et al., 2013).

## Author contributions

PO and MWB conceived the project; PO, MB, EN, and FB carried out analyses; WD, DW, MM, AS, PA contributed samples and reagents; PO wrote the manuscript; MB, EN, FB, and MWB revised the manuscript. All authors approved the final manuscript.

### Conflict of interest statement

The authors declare that the research was conducted in the absence of any commercial or financial relationships that could be construed as a potential conflict of interest.
