# Supplementary material for: Revisiting demographic processes in cattle with genome-wide population genetic analysis
Source: Front Genet. 2015 Jun 2;6:191. doi: 10.3389/fgene.2015.00191 (PMC4451420; doi:10.3389/fgene.2015.00191)
Supplement: Supplementary file 1 [file DataSheet1.DOCX]

**Supporting Material**

SM Table 1. Genes associated to SNPs under selection.

SM Figure 1. Cross-Validation results of the Admixture analysis.

SM Figure 2. Dendrogram showing the clustering between different breeds with bootstrap support values for each branch (Outgroups: OBB, OYK, OGR, OBJ; Ingroups: dark blue (Asian indicine), light blue (African indicine), black hybrids, purple (African taurines), and red (non-Afrian taurine).

SM Figure 3. Plot of residuals from the phylogenetic model without migration shown in Figure 5. Numbers on the margins correspond to the following breeds (abbreviations as in Table1): 1) W_P, 2) CIL, 3) HFD, 4) ANG, 5) HOL, 6) JER, 7) PMT, 8) CHI, 9) ROM, 10) BSW, 11) CHA, 12) NOR, 13) VOS, 14) BAO, 15) LAG, 16) NDA, 17) SOM, 18) BRM.

SM Figure 4. Phylogenetic network of the inferred relationships between 18 cattle breeds. The phylogenetic network inferred by Treemix of the relationships between Welsh White Park cattle breed and 17 other breeds is shown with Brahman (BRM) as outgroup. The figures are from top left to bottom right the same phylogram topology with one migration edge added at the time (top left 1 migration edge, bottom right 8 migration edges). The population lables are: 1) W_P, 2) CIL, 3) HFD, 4) ANG, 5) HOL, 6) JER, 7) PMT, 8) CHI, 9) ROM, 10) BSW, 11) CHA, 12) NOR, 13) VOS, 14) BAO, 15) LAG, 16) NDA, 17) SOM, 18) BRM. The x-axis of each graphs is the drift parameter.

SM Table 1. Protein coding genes associated to SNPs under selection. Genes in close proximity (50,000bp) to the SNPs showing consistent signatures of selection are shown. Genes without known function but only described as “protein coding” gene are not shown. C= chromosome.

| Taurine vs. Indicine comparison | | | | | | | |
| --- | --- | --- | --- | --- | --- | --- | --- |
| Taurine Candidates | | | | Indicine Candidates | | | |
| C | Start | End | Gene | C | Start | End | Gene |
| 2 | 4722619 | 4723281 | SFT2D3 | 2 | 132470750 | 132507576 | HP1BP3 |
| 2 | 65216983 | 65279808 | LYPD1 | 2 | 132522953 | 132529468 | SH2D5 |
| 4 | 29595419 | 29597808 | SP8 | 5 | 25595262 | 25598162 | GLYCAM1 |
| 4 | 70049646 | 70117257 | SNX10 | 5 | 25627052 | 25631242 | PPP1R1A |
| 10 | 13314216 | 13317563 | SNAPC5 | 5 | 25631976 | 25658534 | PDE1B |
| 10 | 13318875 | 13323526 | RPL4 | 8 | 25409464 | 25409555 | Undescribed miRNA |
| 10 | 13320181 | 13320250 | SNORD18 | 9 | 88008820 | 88046821 | LATS1 |
| 10 | 13321373 | 13321470 | SNORD16 | 9 | 88051512 | 88066828 | NUP43 |
| 10 | 13323843 | 13366228 | ZWILCH | 11 | 6721485 | 6757777 | IL1R2 |
| 10 | 13365616 | 13381791 | LCTL | 11 | 6796440 | 6796525 | SNORD86 |
| 10 | 13373031 | 13373162 | SNORA1 | 12 | 10980687 | 10980782 | bta-mir-759 |
| 16 | 37838604 | 37860891 | BLZF1 | 15 | 79187285 | 79188214 | OR4X1 |
| 16 | 37875178 | 37890412 | CCDC181 | 15 | 79208321 | 79209247 | OR4S1 |
| 16 | 73894532 | 73939371 | TRAF5 |  |  |  |  |
| 17 | 70734993 | 70783359 | AP1B1 |  |  |  |  |
| 17 | 70739901 | 70739997 | SNORD125 |  |  |  |  |
| 19 | 43855647 | 43855835 | U2 (snoRNA) |  |  |  |  |
| 19 | 43945992 | 43947907 | ARL4D |  |  |  |  |
| 22 | 39068359 | 39073040 | FEZF2 |  |  |  |  |
| Indicine comparison | | | | | | | |
| Asian Candidates | | | | African Candidates | | | |
| C | Start | End | Gene | C | Start | End | Gene |
| 1 | 1563137 | 1591758 | IL10RB | 1 | 42743866 | 42802957 | DCBLD2 |
| 1 | 1593295 | 1627137 | IFNAR2 | 10 | 77181079 | 77196975 | PLEKHG3 |
| 1 | 10083035 | 10165613 | JAM2 |  |  |  |  |
| 1 | 45489052 | 45521372 | TFG |  |  |  |  |
| 1 | 82176394 | 82210749 | SENP2 |  |  |  |  |
| 1 | 82250680 | 82250794 | 5S_rRNA |  |  |  |  |
| 1 | 82243015 | 82278080 | LIPH |  |  |  |  |
| 1 | 82285913 | 82294596 | TMEM41A |  |  |  |  |
| 1 | 107687596 | 10768817 | ARL14 |  |  |  |  |
| 2 | 113274595 | 11328768 | FAM124B |  |  |  |  |
| 4 | 73367762 | 73368520 | C4H7orf62 |  |  |  |  |
| 5 | 26958461 | 26973297 | ITGB7 |  |  |  |  |
| 5 | 26979339 | 26985686 | ZNF740 |  |  |  |  |
| 5 | 26985263 | 27013078 | CSAD |  |  |  |  |
| 5 | 60225838 | 60226830 | NEUROD4 |  |  |  |  |
| 5 | 60265820 | 60300780 | TESPA1 |  |  |  |  |
| 5 | 27336719 | 27336829 | U6 (snoRNA) |  |  |  |  |
| 7 | 48012507 | 48043848 | CATSPER3 |  |  |  |  |
| 7 | 48063871 | 48069063 | PITX1 |  |  |  |  |
| 7 | 48919355 | 48923271 | IL9 |  |  |  |  |
| 7 | 48966620 | 48985225 | FBXL21 |  |  |  |  |
| 7 | 54624665 | 54639999 | PCDH1 |  |  |  |  |
| 7 | 54670603 | 54685592 | KIAA0141 |  |  |  |  |
| 7 | 54690461 | 54702923 | PCDH12 |  |  |  |  |
| 8 | 25184208 | 25184341 | SCARNA8 |  |  |  |  |
| 8 | 25197704 | 25199286 | RRAGA |  |  |  |  |
| 9 | 45452891 | 45455659 | POPDC3 |  |  |  |  |
| 9 | 45484429 | 45522649 | BVES |  |  |  |  |
| 10 | 7879496 | 7897150 | F2R |  |  |  |  |
| 10 | 32720823 | 32720898 | U4 (snoRNA) |  |  |  |  |
| 11 | 82971327 | 82971429 | 5S_rRNA |  |  |  |  |
| 14 | 31735268 | 31794502 | MTFR1 |  |  |  |  |
| 17 | 46156467 | 46166401 | PUS1 |  |  |  |  |
| 17 | 46171844 | 46193233 | ULK1 |  |  |  |  |
| 18 | 60330959 | 60344819 | ZNF677 |  |  |  |  |
| 19 | 14775262 | 14780081 | CCL14 |  |  |  |  |
| 19 | 14782701 | 14786690 | CCL16 |  |  |  |  |
| 19 | 15233409 | 15265756 | UNC45B |  |  |  |  |
| 19 | 15273798 | 15282574 | NLE1 |  |  |  |  |
| 19 | 15284156 | 15295984 | FNDC8 |  |  |  |  |
| 19 | 15297466 | 15309154 | RAD51D |  |  |  |  |
| 19 | 15308148 | 15308218 | bta-mir-2331 |  |  |  |  |
| 19 | 15406341 | 15425809 | LIG3 |  |  |  |  |
| 19 | 15446157 | 15447896 | ZNF830 |  |  |  |  |
| 19 | 41488358 | 41510910 | SMARCE1 |  |  |  |  |
| 19 | 41515535 | 41524688 | KRT222 |  |  |  |  |
| 19 | 47721711 | 47747246 | MRC2 |  |  |  |  |
| Taurine comparison | | | | | | | |
| European Candidates | | | | Asian Candidates | | | |
| C | Start | End | Gene | C | Start | End | Gene |
| 5 | 108209678 | 108235391 | RAD52 | 2 | 24606880 | 24646776 | HAT1 |
| 10 | 21206754 | 21214476 | JPH4 | 3 | 95367239 | 95367341 | U6 (snoRNA) |
| 10 | 21215969 | 21224329 | AP1G2 | 11 | 55128047 | 55128147 | SNORA72 |
| 10 | 21223286 | 21227253 | THTPA | 11 | 55128047 | 55128147 | SNORA72 |

SM Figure 1.


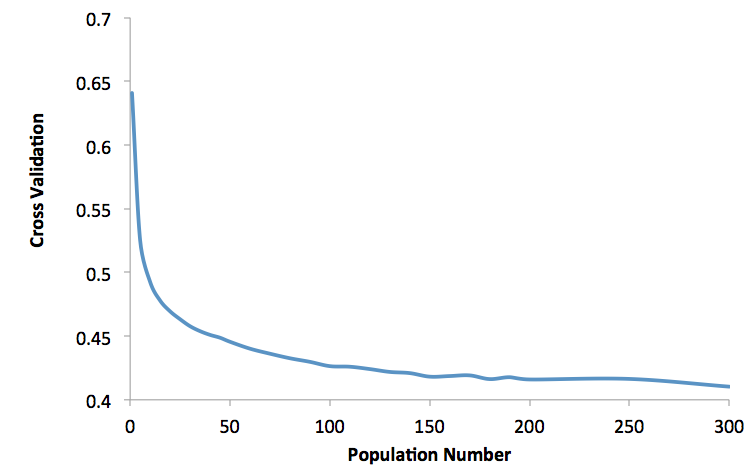


SM Figure 2.


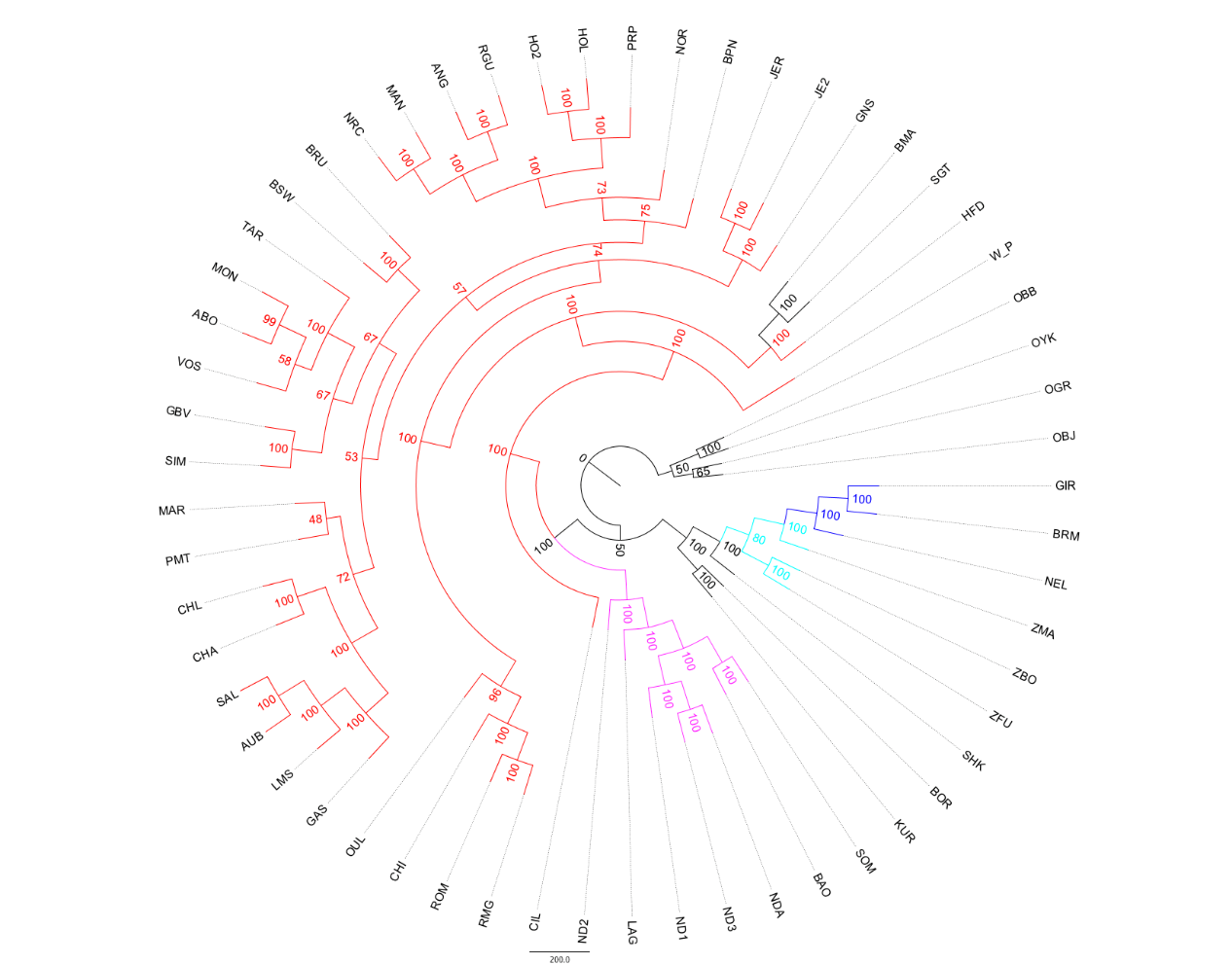


SM Figure 3.

SM Figure 4.
